# Label-free proteomic profiling identifies ECM-related alterations across regeneration states in human peripheral axonal neuropathies

**DOI:** 10.1007/s13105-026-01219-6

**Published:** 2026-08-19

**Authors:** Marina Damato, Soulaimane Aboulouard, Velia La Pesa, Francesco Gentile, Giulia Lunghi, Michela Perrone, Beatrice Pranzo, Elena Chiricozzi, Nilo Riva, Isabelle Fournier, Alessandro Romano, Angelo Quattrini, Michel Salzet, Michele Maffia

**Affiliations:** 1 Dept of Experimental Medicine, Univ. of Salento, Lecce, Italy; 2 Laboratoire Protéomique, Réponse Inflammatoire et Spectrométrie de Masse (PRISM), Universitè de Lille, Inserm, CHU Lille, U1192, Lille, F-59000 France; 3 Neuropathology Unit, Institute of Experimental Neurology, Division of Neuroscience, IRCCS San Raffaele Scientific Institute, Milan, Italy; 4 Department of Medical Biotechnology and Translational Medicine, University of Milano, Segrate, Milano, Italy; 5 3th Neurology Unit and Motor Neuron Disease Centre, Fondazione IRCCS Istituto Neurologico Carlo Besta, Milan, Italy; 6 Department of Life Sciences, Health and Health Professions, Link Campus University, Rome, Italy; 7 CNR NANOTEC – Institute of Nanotechnology, Campus Ecotekne, Lecce, Italy

**Keywords:** Extracellular matrix - ECM, Peripheral nerve, Axonal neuropathy, Protein modulation

## Abstract

Understanding the multiple mechanisms underlying peripheral nerve regeneration in humans is crucial for developing effective therapies for peripheral axonal neuropathies. Although i*n vitro* and in vivo studies have provided significant insights, human studies exploring the composition of proteome remain limited. The ECM plays a crucial role in nerve regeneration, influencing cell adhesion, proliferation, migration, and finally differentiation. In this study, we used a high-sensitivity, label-free liquid chromatography-mass spectrometry (LC-MS) method to investigate the global proteomic profile of human sural nerves aiming to identify molecular dynamics that promote or hinder nerve regeneration in well-characterized cohorts of patients with acute axonal injury, regenerating axonal neuropathy, and non-regenerating axonal neuropathy. Overall, 149 proteins were identified, of which 68 showed significant modulation across the groups. Proteins over-represented in regenerating nerves highlighted the multifactorial nature of regeneration, including immune modulation, debris clearance, cytoskeletal reorganization, axonal extension, and lipid transport. Conversely, structural myelin and neuronal proteins were significantly downregulated in non-regenerating nerves. Among the differentially expressed proteins, functional enrichment analysis revealed a significant overrepresentation of ECM-related components, providing compelling evidence that the ECM is not merely a passive scaffold, but an active driver of peripheral nerve regeneration.

**Supplementary Information:**

The online version contains supplementary material available at 10.1007/s13105-026-01219-6.

## Key Points

ECM remodeling contribute to / influence regenerative outcomes in human axonal neuropathies.

Acutely injured nerves display enrichment in wound-response proteins, consistent with blood–nerve barrier disruption, and formation of a fibrin/fibronectin-rich ECM that supports repair.

Regenerating nerves exhibit a dynamic ECM and a proteomic profile similar to those of controls, reflecting the restoration of a permissive regenerative environment.

Non-regenerating nerves exhibit ECM accumulation and reduced levels of neuronal/myelin structural proteins, consistent with chronic regenerative failure.

**Supplementary Information:**

The online version contains supplementary material available at 10.1007/s13105-026-01219-6.

## Introduction

Peripheral axonal neuropathies may be caused by a variety of metabolic, toxic, genetic and inflammatory disorders. However, in many patients, the etiology remains unknown, such as in idiopathic chronic axonal neuropathy. Peripheral axonal neuropathies present with sensory and/or motor impairment due to progressive axonal loss, leading to frequent severe disability [1–3]. Even if peripheral neurites can regenerate after damage, recovery after axonal degeneration is sometimes inadequate and several patients exhibit progressive worsening without evidence of regeneration. Following nerve damage, the immediate degeneration of the distal nerve stump, referred to as Wallerian degeneration, occurs [4–5]. In the peripheral nervous system (PNS), subsequent axonal regeneration depends on coordinated processes involving immune cells, endothelial cells, fibroblast cells, and Schwann cell proliferation and differentiation, ultimately restoring the nerve tissue structure without fibrosis [4–6]. This nerve regeneration process depends on the interactions between inductive signals from cytokines, growth factors, and extracellular matrix (ECM) components [7–10]. In the peripheral nerve, the ECM includes a specialized basal lamina that surrounds Schwann cells and axons [11–12]. If the basal lamina is disrupted after severe axonal injury, the endoneurium turns into a disorganized mass of Schwann cells, reactive fibroblasts, macrophages and collagen fibers. This disorganization prevents regenerating axons from properly reconnecting to their targets. The complete composition of the human peripheral nerve matrisome and its changes between regenerating and non-regenerating states remain incompletely defined. In this work, we investigated the entire sural nerve proteome using a label-free proteomic approach. We analyzed samples from a carefully characterized cohort of patients experiencing axonal neuropathies with acute axonal injury, active axonal regeneration and chronic axonal damage. Understanding the role of ECM in axonal regeneration may be fundamental for developing therapeutic strategies to prevent axonal degeneration and promote nerve repair in human axonal neuropathies.

## Materials and methods

### Nerve biopsy specimens

Sural nerve biopsy specimens were collected from patients diagnosed with peripheral axonal neuropathy and from control cases. Diagnoses were established through clinical, electrophysiological, and histological examination, including hematoxylin and eosin staining of paraffin sections and analysis of semi-thin sections. All nerve biopsies were obtained with informed consent for diagnostic purposes between December 1994 and April 2002 at the Department of Neurology at Ospedale San Raffaele (Milan, Italy), in accordance with the Declaration of Helsinki. The research has been reviewed and approved by the San Raffaele Ethical Committee. Samples were processed and stored by the institutional biobank Biological Resource Center (CRB-OSR) (Num ID CRB in BBMRI-ERIC: bbmri-eric: ID: IT_1383758011993577). The diagnostic biopsy of the sural nerve was performed under local anesthesia, as described [13]. Each nerve biopsy was divided into three segments: one for routine histology, fixed in 10% formalin and paraffin embedded, a second segment for semi-thin section analysis, fixed in 2% buffered glutaraldehyde, post-fixed in osmium tetroxide (1%), dehydrated and embedded in Epon, and a third segment was immediately frozen in liquid nitrogen. Transverse semi-thin Sect.  (0.5–1 μm) were cut and stained with toluidine blue and examined by light microscope.

Patients were assigned to four groups based on histological findings, which served as unique criteria, as described [14]. Group 1 (Controls): Cases 1–3, exhibiting no pathological evidence of nerve damage; Group 2 (Acute Axonal Damage): Cases 4–6, showing signs of acute axonal damage; Group 3 (Regenerating Axons): Cases 7–9, characterized by active axonal regeneration; Group 4 (Non-Regenerating Axons): Cases 10–12, presenting severe chronic axonal neuropathy without signs of regeneration (Table 1).

**Table 1:** Histopathological diagnosis classified as normal, acute axonal damage, chronic axonal regenerating and chronic axonal non-regenerating

Case Age year Sex	Neuropathy	Histological Diagnosis	FL	Ax De	Ax Re	IC
1 55 M	Control	Normal	1	0	0	0
2 75 M	Control	Normal	0	0	1	0
3 62 F	Control	Normal	0	0	0	0
4 22 M	Acute axonal SM	Acute axonal damage	2	3	0	+
5 75 M	Acute axonal SM	Acute axonal damage	2	2	0	+
6 70 F	Acute axonal SM	Acute axonal damage	1	2	1	+
7 55 M	Chronic axonal SM	Chronic axonal regenerating	2	0	2	0
8 65 F	Chronic axonal SM	Chronic axonal regenerating	1	0	2	0
9 65 M	Chronic axonal SM	Chronic axonal regenerating	1	0	3	0
10 64 F	Chronic axonal SM	Chronic axonal non-regenerating	3	0	0	0
11 62 F	Chronic axonal SM	Chronic axonal non-regenerating	2	0	0	0
12 66 M	Chronic axonal SM	Chronic axonal non-regenerating	3	0	0	0

*M* male, *F* female, *SM* sensory-motor, Fiber loss: (0 = no signs, 1 = mild, 2 = moderate, 3 = severe). Ax De: axonal degeneration (0 = no signs, 1 = mild, 2 = moderate, 3 = severe). Ax Re: clusters of Axonal Regeneration (0 = not present, 1 = low/absent, 2 = moderate, 3 = strong). IC: inflammatory cells infiltration (0 = absent, + = present)

### Sample preparation for mass spectrometry (MS)

Formalin-fixed Paraffin-Embedded (FFPE) blocks were cut into 10 μm sections, with three slides per nerve sample for MS analysis. 

Tissue sections were first deparaffinized by immersing them in two successive baths of 100% xylene for 5 min each. Rehydration was then performed through sequential immersions in the following solutions: two baths of 90% ethanol (5 min each), one bath of 30% ethanol (5 min), and two baths of 10 mM ammonium bicarbonate (NH₄HCO₃, 5 min each). Tissues were subsequently dried under vacuum. Fifty microliters of Tris-HCl buffer (pH 9) were added to each sample, followed by heating at 90 °C for 15 min. Samples were then resuspended by vortexing for 10 s, sonicating for 5 min at 40 °C, vortexing again for 10 s, and briefly centrifuging. Proteins were digested by adding 1 µg of trypsin (20 µg/mL) and incubating overnight at 37 °C. Digestion was stopped by the addition of 5% trifluoroacetic acid (TFA). Samples were then dried under vacuum before further processing.

### LC-MS/MS analysis

Each sample was desalted using Evotip Pure cartridges (Evosep, Denmark) according to the manufacturer’s instructions. Peptides were subsequently separated on an Evosep One liquid chromatography system [15] and analyzed with a Thermo Scientific Q-Exactive Orbitrap mass spectrometer. The system picks up the tip and performs the elution directly in liquid chromatography. The peptides were separated using the C18 endurance column (15 cm x 150 μm ID, 1.9 μm) and the extended method 15 SPD (Sample Per Day). Mobile phase A was 0.1% FA in water and phase B was 0.1% FA in acetonitrile. The mass spectrometer operated in a data-dependent acquisition mode, selecting the ten most intense precursor ions (Top 10) from each MS scan for fragmentation. Full MS survey scans were recorded on the Orbitrap analyzer at a resolution of 70,000 (FWHM), over a mass range of m/z 300–1600, with an AGC target of 3 × 10⁶ and a maximum injection time of 120 ms. MS/MS spectra were acquired over an m/z range of 200–2000 with a resolution of 17,500 (FWHM), an AGC target of 1 × 10⁵, and a maximum injection time of 60 ms. Fragmentation was performed by Higher Energy Collisional Dissociation (HCD) with a normalized collision energy of 30%. Only precursor ions with charge states between + 2 and + 7 were selected for fragmentation, and dynamic exclusion was set to 20 s.

MS raw files were processed using the MaxQuant proteomics software (version 1.6.3.4) [16–17] and peptides were searched against the *Homo sapiens* UniProt protein database (Release February 2022, 79052 entries) using the Andromeda algorithm [18]. Trypsin was used as the proteolytic enzyme, allowing up to two missed cleavages. Methionine oxidation was specified as a variable modification. For the MS spectra, an initial mass tolerance of 6 ppm was applied, and a fragment mass tolerance of 20 ppm was used for the HCD MS/MS data. The False Discovery Rate (FDR) was set to 1% for the identification of both peptides and proteins. Protein quantification was performed using a label-free, relative quantification approach, employing the MaxLFQ algorithm [19].

Label-free experiments were performed in biological triplicates, except for two control subjects, in which the third sample failed during technical preparation, leaving no proteomic data. Consequently, analyses for these two cases were conducted in duplicate.

Statistical analysis was performed with the Perseus software (v.1.6.2.1) [17]. Proteins detected in the reverse decoy database, classified as potential contaminants, and identified solely through site-specific modifications, were excluded from the matrix data. Data were log2-transformed and further filtered to retain proteins expressed in at least 70% of one group for each condition considered. To identify proteins with significant differential expression across the four experimental groups, a multiple-sample ANOVA test was performed (*p* ≤ 0.05). Pairwise comparisons between the control group (Group 1) and each pathological group were subsequently conducted using Student’s t-tests (*p* ≤ 0.01) as a post hoc exploratory analysis to facilitate the interpretation of the specific differences contributing to the global ANOVA results. To control for multiple hypothesis testing, the false discovery rate (FDR) was adjusted using the Benjamini–Hochberg procedure where appropriate. Missing values were imputed using a normal distribution with the imputation function. LFQ intensities were then z-scored and subjected to hierarchical clustering using Euclidean distance as a measure for both column and row. The robustness of the identified protein expression patterns was further supported by the consistency observed across eight biological replicates, as well as by hierarchical clustering, principal component analysis (PCA), and independent validation experiments. The STRING database (v. 12.0) was employed to perform functional annotation and characterization of the resulting protein networks [20], applying a false discovery rate (FDR) threshold of 5% and a required interaction score set to medium confidence (0.400).

### Cell culture and reagents

RT4-D6P2T cells (ATCC Catalog No. CRL-2768) were maintained in Dulbecco’s Modified Eagle’s Medium (4500 mg/L glucose, EuroClone, Milan, Italy) supplemented with 10% FBS, 100 U/mL penicillin, 100 µg/mL streptomycin and 2 mmol/L glutamine at 37 ° C in an atmosphere of 5% CO2. For experimental treatments, culture vessels were pre-coated with fibronectin (10 µg/mL; Cat. No. F4759, Sigma-Aldrich) prior to cell seeding. Cells were then cultured on the coated surfaces and treated for 48 h.

### Immunofluorescence analysis

To verify the protein levels by immunofluorescence analysis in nerve biopsies, Cofilin was selected based on its functions and the extent of expression between the groups. Double immunofluorescence was performed on sural nerve from patients with axonal neuropathies and controls using antibodies to Cofilin-1, phosphorylated Cofilin (p-Cofilin), and myelin protein zero (MPZ) to mark myelin. Controls included omission of primary antibodies on parallel sections. Slides were incubated overnight at 4 °C with primary antibodies against Cofilin (1:50; Santa Cruz; sc-376476) and MPZ (1:100; Invitrogen; code: 10572-1-AP), as well as p-Cofilin (1:100; Santa Cruz; sc-365882) and MPZ. Sural nerve sections were dewaxed, rehydrated, washed in 0.1 M PBS, and blocked in 5% bovine serum albumin and 3% goat serum. After washing, sections were incubated with Alexa 488- and 546-conjugated secondary antibodies (1:1000; Invitrogen) for 1 h at room temperature, then counterstained with DAPI. Sections were mounted with Fluoroshield™ (Sigma-Aldrich) and analyzed using a confocal laser-scanning microscope (LSM700, Zeiss) with a 40× oil-immersion objective. All slides were examined by experienced neuropathologists, who were blinded to the pathological data.

Immunofluorescence analysis in cell culture experiments. RT4-D6P2T cells were fixed with 4% paraformaldehyde in PBS for 15 min, permeabilized with 0.2% Triton-X100 (Sigma Aldrich) in PBS for 15 min, and blocked for 30 min in 5% bovine serum albumin (BSA, Sigma Aldrich) in PBS. Samples were then incubated overnight at 4 °C with primary antibody (p-Cofilin, 1:50, Santa Cruz Biotechnology) in blocking solution. After washing, cells were incubated with appropriate Alexa Fluor 594 secondary antibody (1:1000, Invitrogen) and FITC-conjugated phalloidin for 1 h at room temperature, then counterstained with DAPI. Cells were mounted with Fluoroshield™ (Sigma-Aldrich) and analyzed using a confocal laser-scanning microscope (LSM700, Zeiss) with a 20× objective.

### Western blot analysis

Cell lysates were extracted in RIPA buffer (Cell Signaling) and quantified by BRADFORD method (BIORAD, Hercules, CA, USA). About 20 µg of proteins were mixed 1:1 with Laemli buffer (SIGMA, St. Louis, MO, USA), boiled for 5 min, separated by 12% SDS-PAGE, and transferred to the Hybond ECL nitrocellulose membrane (GE Healthcare, Chicago, IL, USA). Subsequently, membranes were blocked for 1 h in Blotto A, (Santa Cruz, CA, USA) at room temperature, and incubated for 1–2 h at room temperature with a primary antibody diluted in Blotto A. After two 10-minute washes with TBST (10 mM Tris, pH 8.0, 150 mM NaCl, 0.5% Tween 20), membranes were incubated with HRP-conjugated secondary antibodies for 1 h at room temperature. Membranes were washed twice for 5 min with TBST, and signals were then developed using the Amersham ECL western blotting detection system (GE Healthcare, Chicago, IL, USA) and ChemiDoc System (Bio-Rad). Densitometric analysis was performed using Image Lab software (Bio-Rad) to determine the optical density (OD) of the bands. Densitometric analysis of the bands was performed using Image Lab software (version 6.0.1, Bio-Rad Laboratories, Hercules, CA, USA).

Membranes were probed with the following primary antibodies (1:1000 dilution): p-Cofilin (Santa Cruz; sc-365882), Cofilin (Santa Cruz; sc-23779), GAPDH (mAbcam 9484); Anti-β-Actin−Peroxidase antibody (Sigma Aldrich #A38549) (1:20000 dilution). Secondary antibodies (HRP-conjugated) were from Invitrogen (1:10.000 dilution) (goat anti-Rabbit IgG (H + L) Secondary Antibody, HRP #31460; Goat anti-Mouse IgG (H + L) Highly Cross-Adsorbed Secondary Antibody, Alexa Fluor™ 488 #A11029).

## Results

### Histological grouping of human sural nerve biopsies

**Fig. 1:**
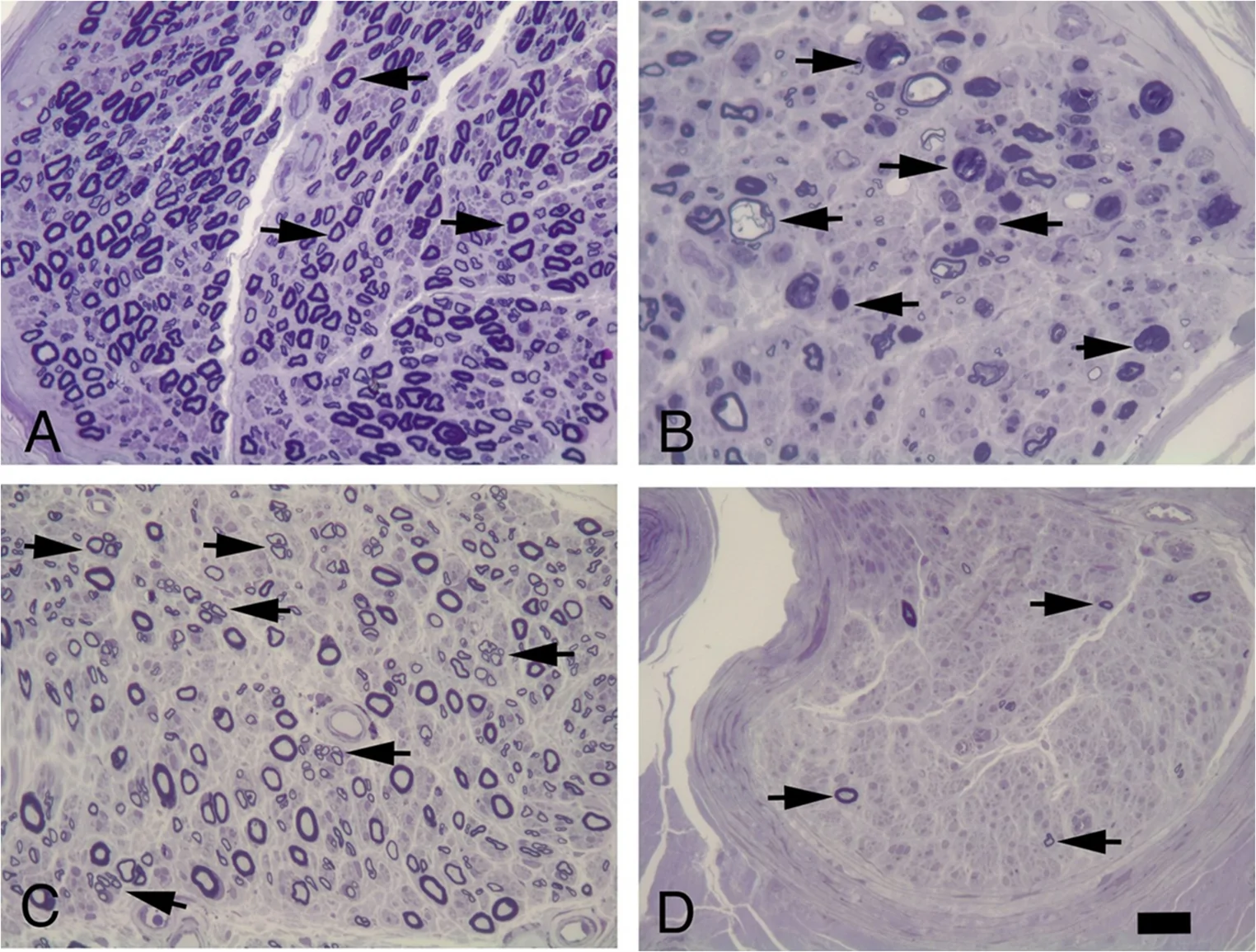
Histopathological findings of the human sural nerve biopsies. Representative semithin cross sections of the sural nerve biopsies stained with Toluidine blue for light microscopy. Group 1 (**A**): Normal sural nerve showing the endoneurium that contains normal numbers of small and large myelinated nerve fibers (**A**, arrows) without signs of axonal degeneration and regeneration. Group 2 (**B**): Acute axonal neuropathy, many ovoids indicating axonal degeneration (arrows) are present, without axonal regeneration. Group 3 (**C**): Chronic axonal neuropathy with regeneration. A mild reduction of large, myelinated nerve fibers is present. Arrows in **C** indicate several closely joined thin myelinated axons that represent “regenerating clusters”, indicating successful axonal regeneration after axonal damage. Group 4 (**D**): Severe chronic axonal neuropathy, semithin cross sections show severe reduction of myelinated nerve fibers, endoneurial fibrosis, and a few residual myelinated fibers are present (**D**, arrows). Bar: 20 μm

A total of 12 patients who underwent sural nerve biopsy were recruited and grouped according to histological findings. Group 1 (control, cases 1–3) displayed nerves within normal limits (Fig. 1A). Group 2 (cases 4–6) showed a reduction of myelinated nerve fibers and abundant ovoids, indicative of acute axonal degeneration (Fig. 1B). In Group 3 (cases 7–9), a remarkable increase in clusters of thinly myelinated fibers was observed, suggesting active axonal regeneration (Fig. 1C). Conversely, Group 4 (cases 10–12) exhibits severe loss of myelinated nerve fibers with very few myelin-forming units remaining and a marked increase in endoneurial connective tissue (Fig. 1D).

### Proteomic analysis of formalin-fixed-paraffin-embedded human sural nerves

Proteomic analysis of the nerve samples identified 149 proteins, of which 68 were significantly modulated and grouped into four clusters (Table 2).

**Table 2:** Protein clusters showing differential expression between patient groups as identified by LC-MS/MS analysis

**Cluster 1**		
**Protein IDs**	**Protein names**	**Gene names**
Q05707-2	Collagen alpha-1(XIV) chain	COL14A1
H7BXV5	Collagen alpha-1(XVIII) chain; Endostatin	COL18A1
P12111-2	Collagen alpha-3(VI) chain	COL6A3
P12110	Collagen alpha-2(VI) chain	COL6A2
P12109	Collagen alpha-1(VI) chain	COL6A1
A0A6Q8PFJ0	Prelamin-A/C; Lamin-A/C	LMNA
P51888	Prolargin	PRELP
P02461	Collagen alpha-1(III) chain	COL3A1
**Cluster 2**		
**Protein IDs**	**Protein names**	**Gene names**
Q16777	Histone H2A type 2-C	HIST2H2AC
P08758	Annexin A5	ANXA5
P12036-2	Neurofilament heavy polypeptide	NEFH
P08133-2	Annexin A6;Annexin	ANXA6
A0A1C7CYX9	Dihydropyrimidinase-related protein 2	DPYSL2
P51911	Calponin-1;Calponin	CNN1
P18206-2	Vinculin	VCL
P55268;F5H520	Laminin subunit beta-2	LAMB2
A0A2R8YGX3	Tropomyosin alpha-4 chain	TPM4
A0A0G2JIW1	Heat shock 70 kDa protein 1 A	HSPA1A
**Cluster 3**		
**Protein IDs**	**Protein names**	**Gene names**
Q2UY09	Collagen alpha-1(XXVIII) chain	COL28A1
K7ERX7	ATP synthase subunit alpha, mitochondrial	ATP5A1
H3BPS8	Fructose-bisphosphate aldolase	ALDOA
Q71U36-2	Tubulin alpha-1 A chain	TUBA1A
P41219	Peripherin	PRPH
Q13885	Tubulin beta-2 A chain	TUBB2A
P07196	Neurofilament light polypeptide	NEFL
A0A5F9ZI26	Myelin protein P0	MPZ
P09543-2	2,3-cyclic-nucleotide 3-phosphodiesterase	CNP
E7EMV2	Neurofilament medium polypeptide	NEFM
P02689	Myelin P2 protein	PMP2
J3QL64	Myelin basic protein	MBP
A0A669KBF1	Periaxin	PRX
P11142-2	Heat shock cognate 71 kDa protein	HSPA8
P00505-2;P00505	Aspartate aminotransferase, mitochondrial	GOT2
P68371	Tubulin beta-4B chain	TUBB4B
A0A6I8PIT5	Heat shock 70 kDa protein 12 A	HSPA12A
**Cluster 4**		
**Protein IDs**	**Protein names**	**Gene names**
P02787	Serotransferrin	TF
P06576	ATP synthase subunit beta, mitochondrial; ATP synthase subunit beta	ATP5B
P26038	Moesin	MSN
P62805	Histone H4	HIST1H4A
P08670	Vimentin	VIM
P04083	Annexin A1;Annexin	ANXA1
E9PP50	Cofilin-1;Cofilin-2	CFL1;CFL2
P02751-5	Fibronectin	FN1
C9JF17	Apolipoprotein D	APOD
A0A7P0Z4C6	Transitional endoplasmic reticulum ATPase	VCP
A0A7I2V4I6	Heterogeneous nuclear ribonucleoproteins A2/B1	HNRNPA2B1
Q5TEC6	Histone H3	HIST2H3PS2
P02743	Serum amyloid P-component	APCS
A0A286YEY1	Ig alpha-1 chain C region	IGHA1
P06727	Apolipoprotein A-IV	APOA4
P02649	Apolipoprotein E	APOE
P01024	Complement C3	C3
Q5T985	Inter-alpha-trypsin inhibitor heavy chain H2	ITIH2
P19827	Inter-alpha-trypsin inhibitor heavy chain H1	ITIH1
F5GXS0	Complement C4-B	C4B
U3KQK0	Histone H2B	HIST1H2BN
P35579	Myosin-9	MYH9
P08294	Extracellular superoxide dismutase [Cu-Zn]	SOD3
P63104	14-3-3 protein zeta/delta	YWHAZ
P09211	Glutathione S-transferase P	GSTP1
P02730	Band 3 anion transport protein	SLC4A1
P61978-3	Heterogeneous nuclear ribonucleoprotein K	HNRNPK
P01023	Alpha-2-macroglobulin	A2M
P02671-2	Fibrinogen alpha chain	FGA
I3L4N8	Actin, cytoplasmic 2	ACTG1
F8W1R7	Myosin light polypeptide 6	MYL6
P0DP25	P0DP25	CALM2
A0A0A0MS08	Ig gamma-1 chain C region	IGHG1

**Fig. 2:**
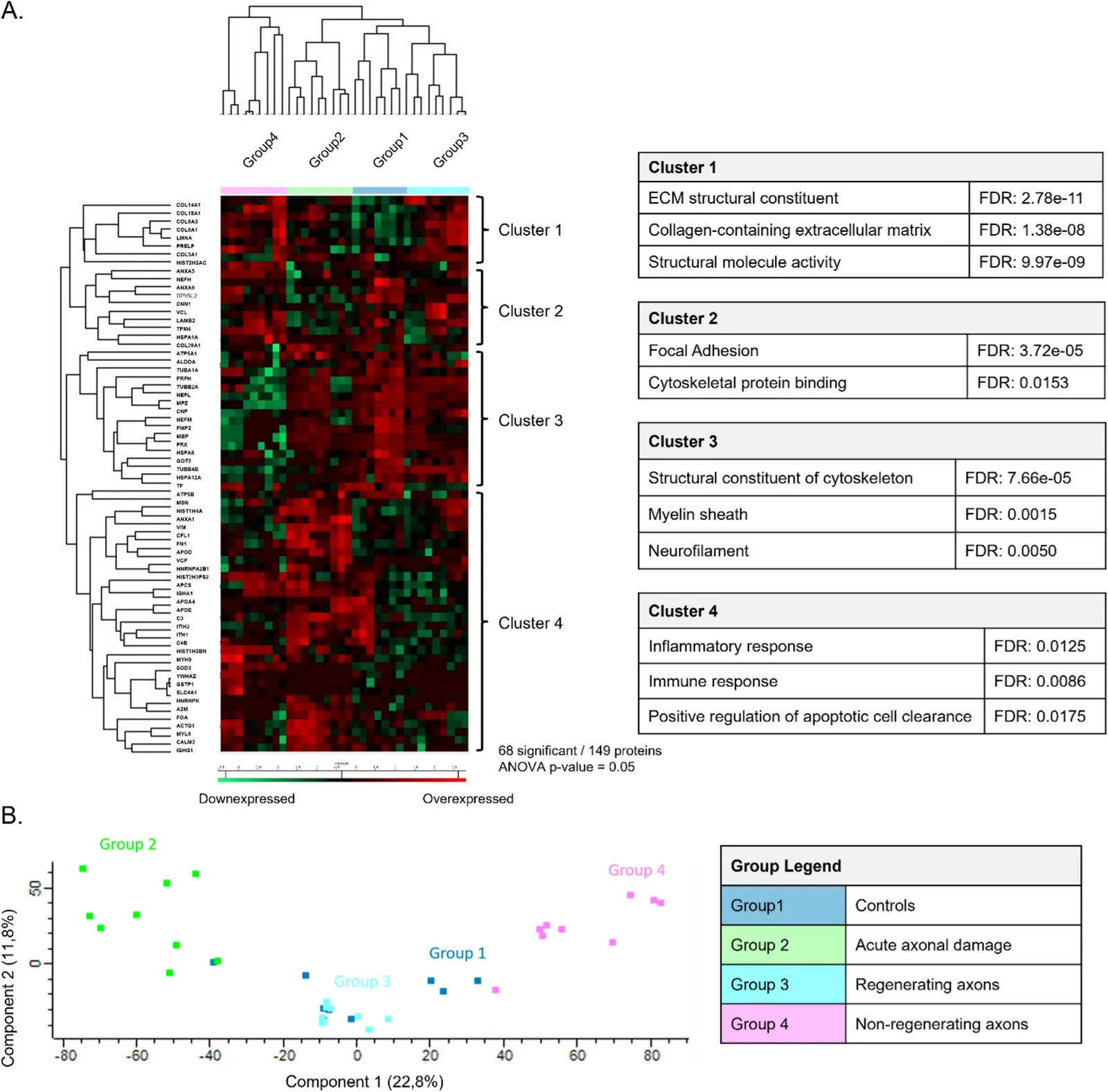
Proteomic analysis of sural nerve biopsies. (**A**) The heat map based on Euclidean distance showed a significant separation between the groups of collected samples. The color scale ranges from red to green (highest to lowest relative expression). Each column of the heat map represents an independent sample and each row represents a specific protein. Clusters of proteins modulated across different conditions are highlighted by curly brackets. The results of the Gene Ontology (GO) analysis, performed using the STRING database (v12.0), are shown in the tables. (**B**) Principal Component Analysis (PCA) showing the distribution based on the first two principal components. The plot illustrates the clustering and separation of samples based on experimental conditions, indicating differences in their global expression profiles

A multiple sample test (ANOVA, p-value = 0.05) and hierarchical clustering based on Euclidean distance showed a clear separation among the groups, with Group 4 (non-regenerating axons) clustering on a distinct branch compared to the others, accordingly with its unique histological features characterized by severe depletion of nerve fibers (Fig. 2A).

Cluster 1 contained 8 proteins overexpressed in pathological conditions (Groups 2–4). This network primarily included ECM proteins such as COL14A1, COL18A1, COL6A1, COL6A2, COL6A3, COL3A1, PRELP, and the nuclear protein LMNA, indicating enhanced ECM activity in nerve damage and regeneration, underscoring the ECM’s active role in these processes. Cluster 2 comprised 10 proteins, which were mainly downregulated in Group 2 and overexpressed in the other groups. GO term enrichment analysis performed by STRING database (v.12) identified Cellular Components related to Focal Adhesion (FDR: 3.72e-03) and Molecular Functions associated with Cytoskeletal Protein Binding (FDR: 0.0153). Cluster 3 included 17 proteins that were downregulated in Group 4 and overexpressed in the other groups. This cluster was characterized by proteins involved in the structural functions of myelin and neurons. The STRING analysis revealed a highly interconnected network, particularly among myelin-associated proteins (MPZ, PMP2, MBP, CNP, PRX), cytoskeletal compounds (TUBB2A, TUBB4B, NEFL, NEFM) and stress response proteins (HSPA8, HSPA12A). This suggests a coordinated effort in maintaining neuronal structure and function, as well as a response to pathology that failed in the severe, chronic conditions of Group 4. Cluster 4, the largest group, contained proteins that were globally overrepresented in Group 2. This cluster highlighted the multifactorial nature of peripheral nerve regeneration, involving cytoskeletal reorganization, axonal extension, immune modulation, debris clearance, ECM remodeling and lipid transport. STRING analysis identified highly represented Biological Processes related to Acute-phase response (FDR:0.0086), Response to wounding (FDR:0.0071), Lipid transport (FDR: 0.0130), Inflammatory response (FDR:0.0125), Immune response (FDR: 0.0086), Response to reactive oxygen species (FDR: 0.00027), Cytoskeleton organization (FDR: 0.0462), and Regulation of cellular component organization (FDR: 0.00041).

Principal component analysis (PCA) further revealed variability among the experimental groups (Fig. 2B).

Group 3 exhibited a molecular profile comparable to that of controls (Group 1), according to the active axonal regeneration observed in these nerves. On the other hand, Groups 2 and 4 did not cluster together or with Groups 1 and 3, indicating that distinct molecular profiles characterize nerves with acute axonal degeneration (Group 2) or non-regenerating nerves (Group 4).

### Pairwise Proteomic Comparisons and Functional Insights

We then compared the control nerves with the pathological groups. Pairwise comparisons using Student’s t-test (p-value = 0.05) revealed significant differences between the groups.

**Fig. 3:**
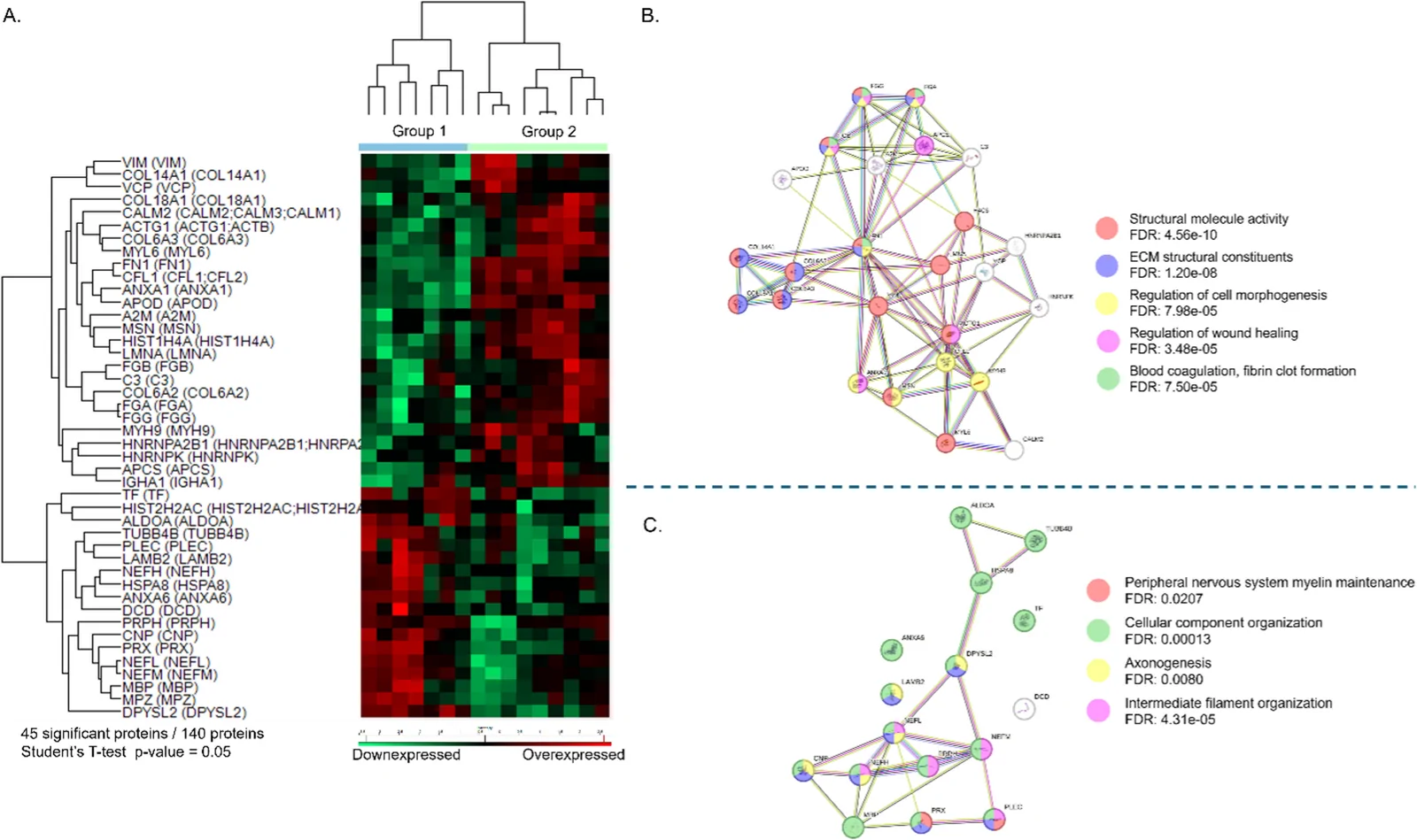
Proteomic analysis of sural nerve biopsies: comparison between group 1 and group 2. **A**. The heat map, based on Euclidean distance, showed a significant separation between Group 1 and Group 2. This analysis identified two protein clusters that were significantly modulated. **B**, **C**. Both clusters were subjected to STRING (v12.0) analysis, and the resulting molecular interaction networks, along with the GO enrichment analysis, are shown in the figure

Comparing Control (Group 1) with Acute Axonal Damage (Group 2) (Fig. 3A), we identified significantly modulated Molecular Functions and Biological Processes.

**Fig. 4:**
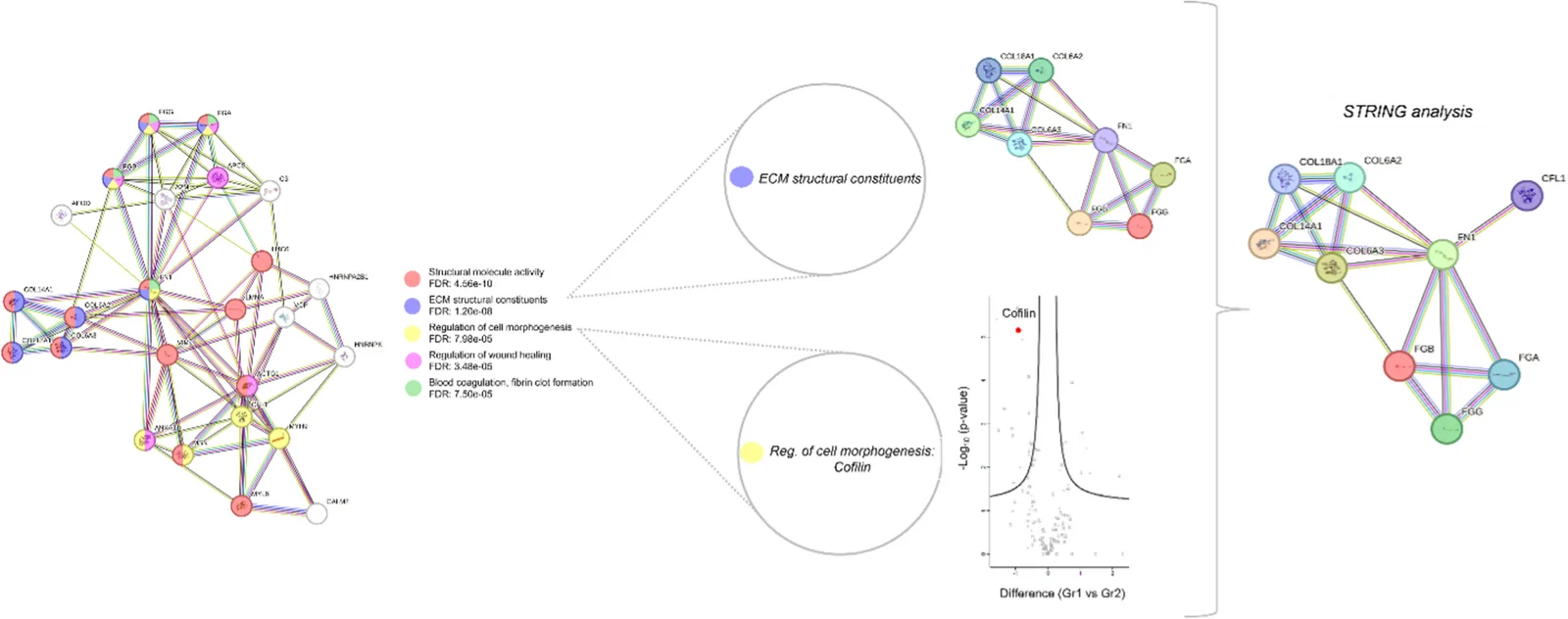
Protein–protein interaction network generated using the STRING database. The figure displays predicted functional associations between proteins of interest, with a focus on “ECM structural constituents” (GO term) and cofilin. The network was constructed using STRING (v12.0). Line thickness indicates interaction confidence, while colored nodes represent upregulated proteins in experimental group 2 compared to the controls. Notably, FN1 appears as a central node with direct interactions, suggesting its potential regulatory role within the network

Structural molecule activity (FDR: 4.56e-10) and ECM structural constituent (FDR: 1.20e-08) were significantly upregulated in Group 2 (Fig. 3B). ECM structural constituents included collagens (COL6A3, COL18A1, COL6A2, COL14A1), fibrinogens (FGG, FGA, FGB), and fibronectin (FN1). Biological Processes upregulated in Group 2 involved Blood coagulation, Fibrin clot formation (FDR: 7.50e-05), Cell adhesion (FDR: 1.45e-05), and Regulation of cell morphogenesis (FDR: 7.98e-05). Conversely, downregulated in Group 2, were primarily associated with Molecular Function linked to Structural constituent of cytoskeleton (FDR: 1.17e-06) and Biological Processes such as Neurofilament bundle assembly (FDR: 4.58e-05), Intermediate filament organization (FDR: 4.31e-05), PNS myelin maintenance (FDR: 0.0207), Schwann cell development (FDR: 0.0044), PNS myelin maintenance (FDR: 0.0207), and Axonogenesis (FDR: 0.0080) (Fig. 3C). Collectively, these observations suggest a pro-inflammatory state in Group 2, characterized by fibrinogen enrichment. Furthermore, an increase in Cofilin activity is observed during acute injury response, supporting actin cytoskeleton remodeling and axonal growth. Notably, a molecular network was generated by submitting all proteins classified as “ECM structural constituents” to the STRING platform, along with cofilin. Within this network, a direct interaction between cofilin and fibronectin is suggested (Fig. 4).

**Fig. 5:**
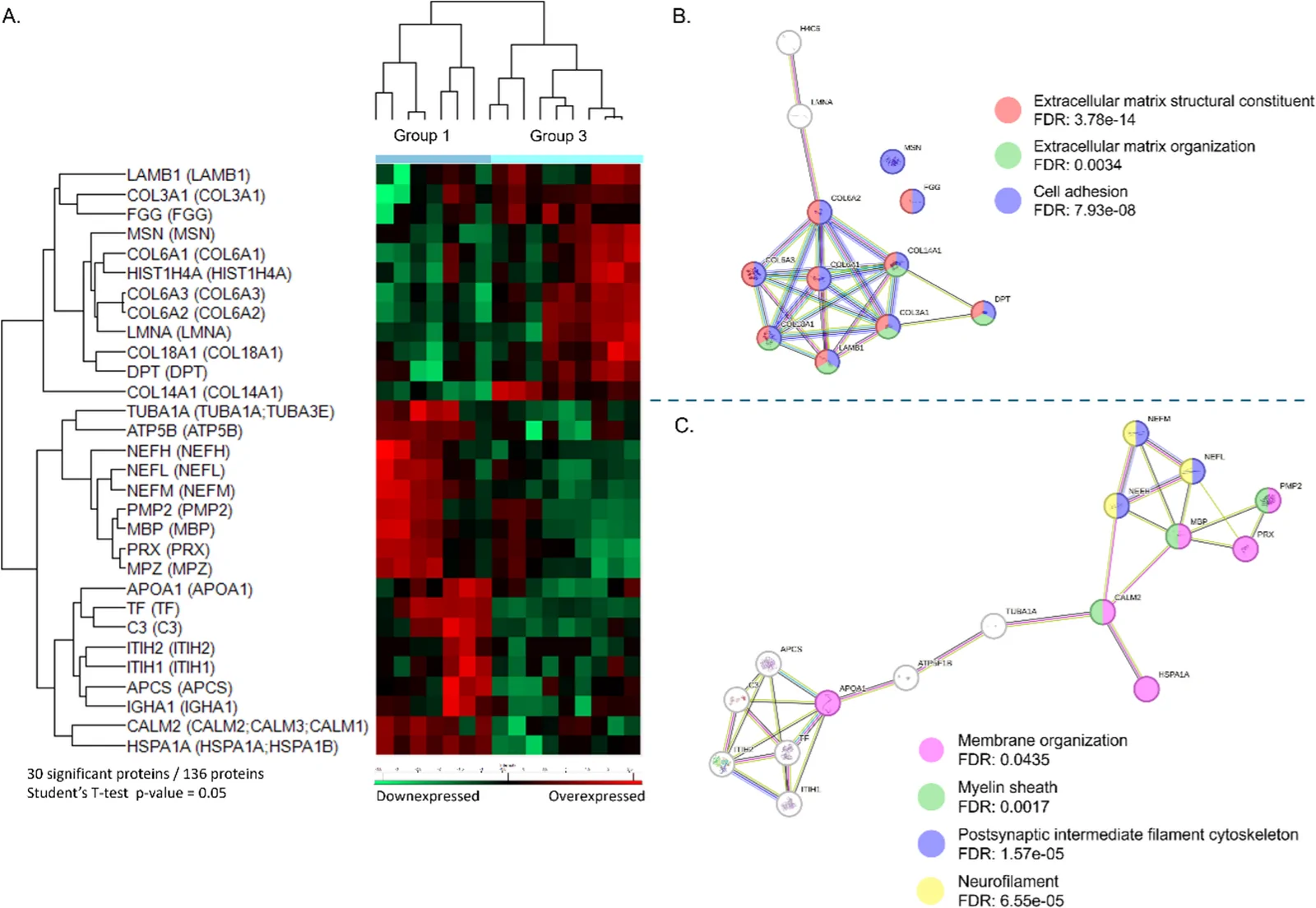
Proteomic analysis of sural nerve biopsies: comparison between group 1 and group 3. **A**. The heat map, based on Euclidean distance, showed a significant separation between Group 1 and Group 3. This analysis identified two clusters of proteins that were significantly modulated. **B**-**C**. Both clusters were subjected to STRING (v12.0) analysis, and the resulting molecular interaction networks, along with the GO enrichment analysis, are shown in the figure

A comparison of the Control (Group 1) and Regenerating Axons (Group 3) revealed that the expression levels of 30 proteins were significantly modulated (Fig. 5A).

The cluster upregulated in Group 3 was characterized by 12 proteins forming a molecular network primarily involved in ECM remodeling and contributing to the regenerative process. STRING analysis highlighted the ECM structural constituent (FDR: 3.78e-14) as a key Molecular Function and Cell adhesion (FDR: 7.93e-08) and ECM organization (FDR: 0.0034) as prominent Biological Process (Fig. 5B). In contrast, the 16 proteins upregulated in Group 1 pertained to Cellular Components such as Postsynaptic intermediate filament cytoskeleton (FDR: 1.57e-05), Myelin sheath (FDR: 0.0017), Neurofilament (FDR: 6.55e-05) and Biological Process of Membrane organization (FDR: 0.0435) (Fig. 5C).

**Fig. 6:**
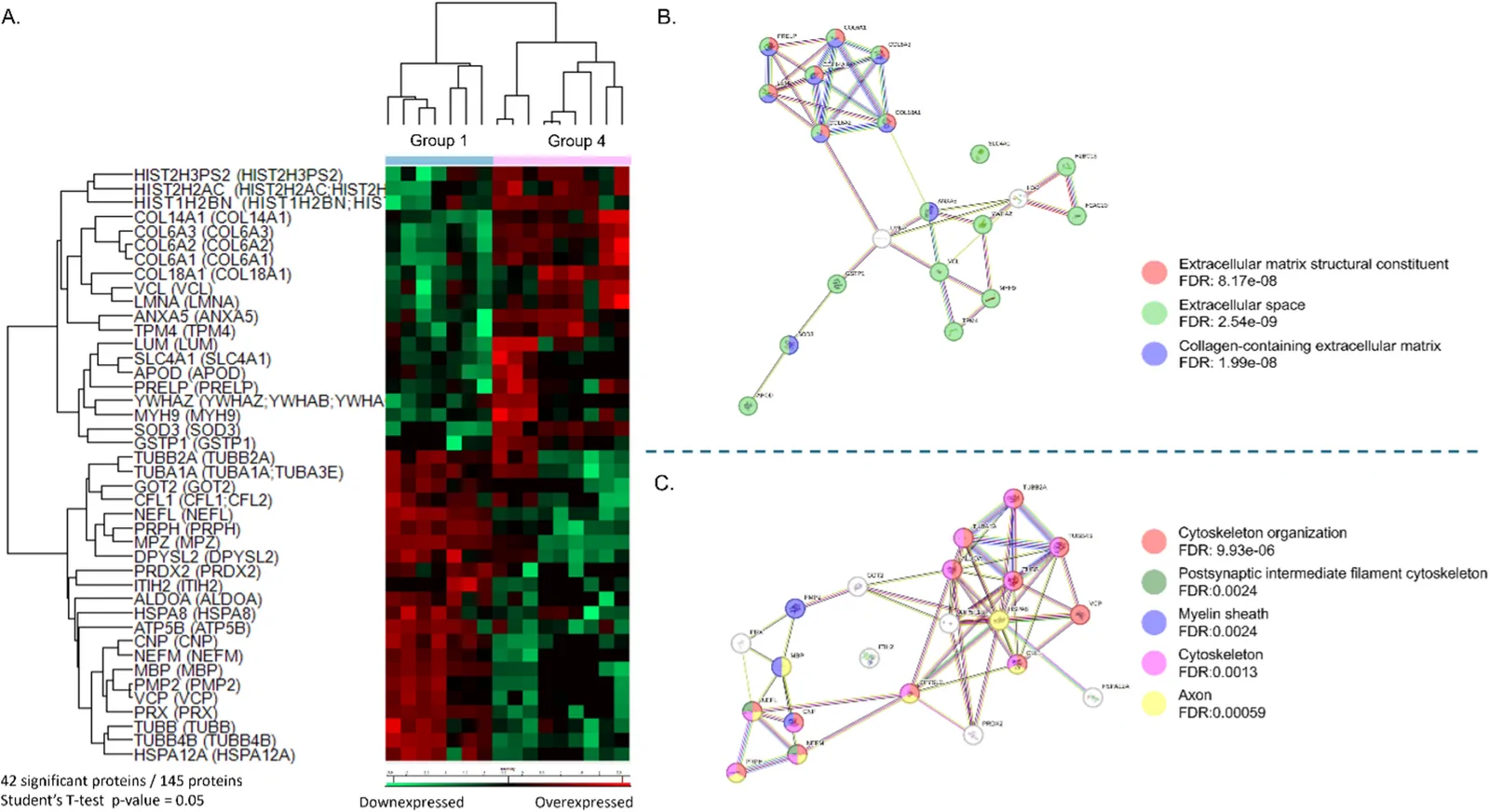
Proteomic analysis of sural nerve biopsies: comparison between group 1 and group 4. **A**. The heat map, based on Euclidean distance, showed a significant separation between Group 1 and Group 4. This analysis identified two clusters of proteins that were significantly modulated. **B**-**C**. Both clusters were subjected to STRING (v12.0) analysis, and the resulting molecular interaction networks, along with the GO enrichment analysis, are shown in the figure

Lastly, comparing Control (Group 1) with Non-Regenerating Axons (Group 4), 42 proteins were identified as significantly modulated and organized into two distinct clusters based on their differential expression (Fig. 6A). The cluster upregulated in Group 4 (21 proteins) again highlighted ECM structural constituent (FDR: 8.17e-08) as the most represented Molecular Function. Cellular components such as Extracellular space (FDR: 2.54e-09) and Collagen-containing ECM (FDR: 1.99e-08) were also highly significant (Fig. 6B).

### Immunofluorescence validation of human sural nerve sections

**Fig. 7:**
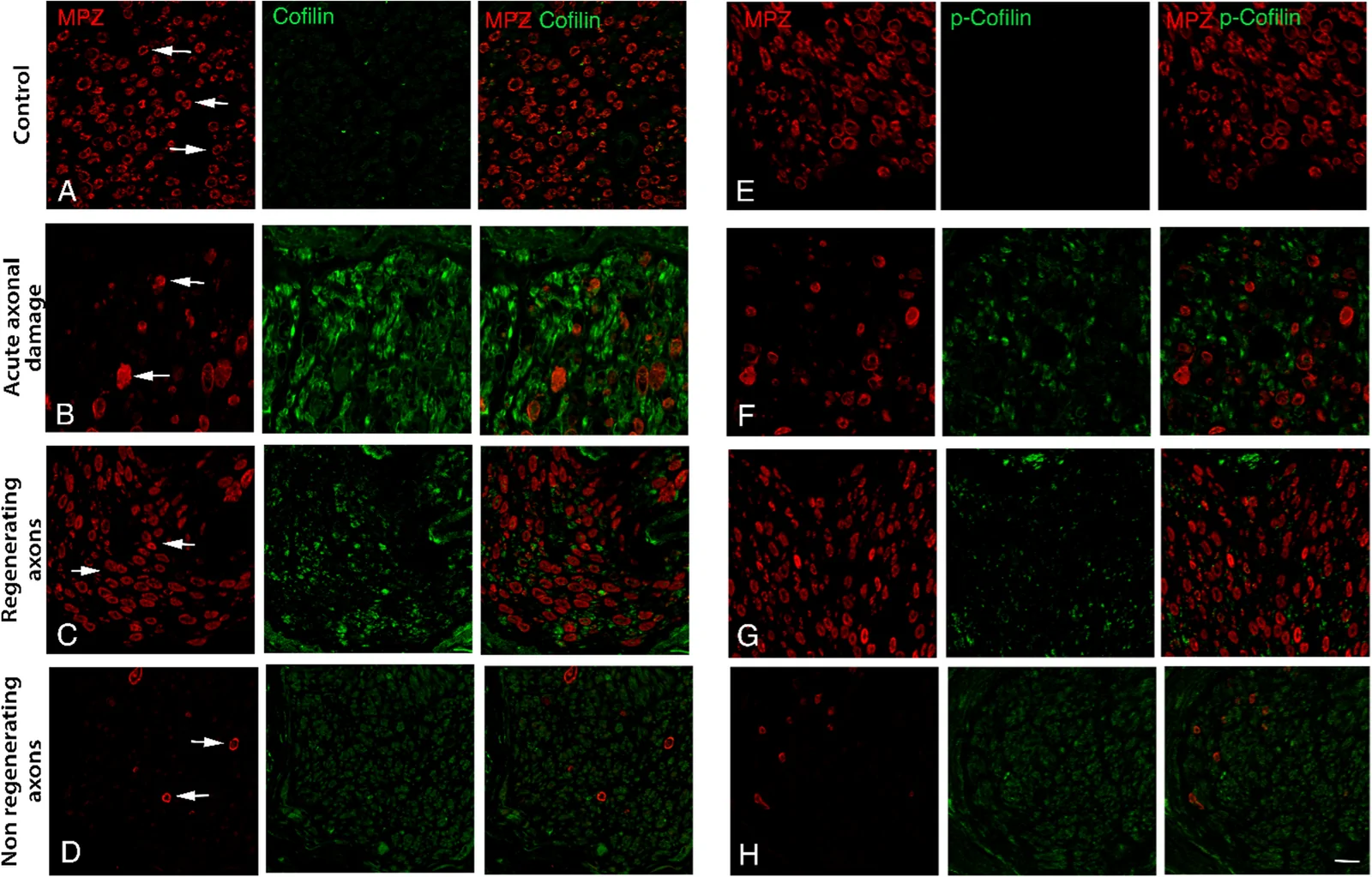
Different expressions of Cofilin and p-Cofilin in patients with axonal neuropathies. Transverse sections of control (**A** and **E**), acute axonal (**B **and **F**), regenerating (**C **and **G**), and non-regenerating sural nerve (**D **and **H**). In control nerves, double staining for MPZ (myelin protein zero) shows high positivity of typical myelinated nerve fibers (**A**, arrows). Cofilin expression was almost undetectable. In cases of acute axonal neuropathy, disrupted staining for MPZ (**B**, arrows) and high positivity for Cofilin in the endoneurium (**B**). Moderate expression of Cofilin in the endoneurium was observed in regenerating nerves (**C**), MPZ-stained myelin sheaths (**C**, arrows). In contrast, Cofilin expression was very low in non-regenerating nerves (**D**), with few residual myelinated nerve fibers (**D**) positive for MPZ. The expression patterns of p-Cofilin were similar to those of Cofilin, with undetectable levels in control nerves (**E**), moderate expression in acute axonal neuropathy (**F**), and minimal expression in regenerating nerves (**G**) and non-regenerating nerves (**H**). Bar: 20 μm

Immunofluorescence was performed to validate proteomic results, specifically by detecting Cofilin in human sural nerves, which was found to interact directly with fibronectin. In control nerves, Cofilin expression was almost undetectable (Fig. 7A), indicating minimal or no active actin remodeling in the absence of injury. In cases of acute axonal neuropathy, Cofilin expression was strong (Fig. 7B), suggesting a robust activation of actin dynamics in response to injury. Moderate expression of Cofilin was observed in regenerating nerves (Fig. 7C), probably reflecting a controlled activation of the cytoskeleton remodeling process. In contrast, Cofilin expression was very low in non-regenerating nerves, indicating minimal cytoskeletal remodeling (Fig. 7D). The expression patterns of p-Cofilin were similar to Cofilin, with undetectable levels in control nerves (Fig. 7E), moderate expression in acute axonal neuropathy (Fig. 7F), and regenerating nerves (Fig. 7G), and low expression in non-regenerating nerves (Fig. 7H), highlighting the importance of Cofilin phosphorylation in regulating axonal growth and regeneration.

To functionally validate the mechanistic link between the ECM protein signature identified by proteomics and modulation of the cofilin-actin axis in nerves, an immortalized Schwann cell line (RT4-D6P2T) was used as a model for nerve glial cells. RT4-D6P2T cells were cultured on fibronectin-coated plates (10 µg/ml) and compared with matched uncoated controls at 48 h. Fibronectin was selected as the ECM stimulus because it showed significant modulation in our proteomic data and serves as the central node linking the modulated ECM proteins to cofilin. Moreover, it is known to play a role in peripheral nerve repair.

**Fig. 8:**
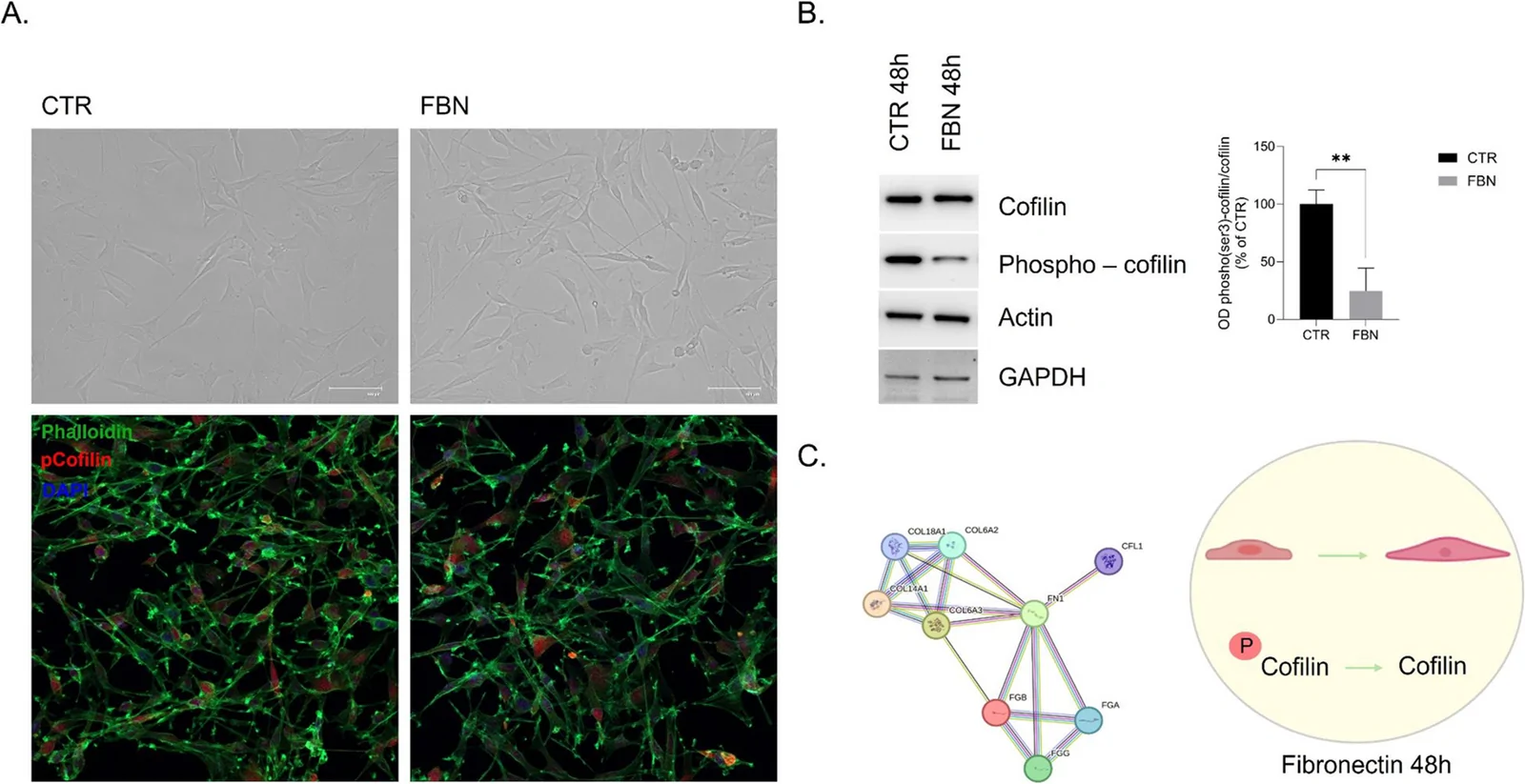
Morphological changes and cofilin activation induced by fibronectin treatment. (**A**) Upper panels. Representative phase contrast images of RT4D6P2T cells cultured in normal conditions (left) or treated with 10 µg/ml fibronectin (right) for 48 h. Images were acquired using an EVOS Floid Cell Imaging Station (Life Technologies, Carlsbad, CA, USA) equipped with a 20X objective. Scale bar 100 μm. Lower panels. Representative confocal images of RT4-D6P2T cells cultured in normal conditions (CTR) and on fibronectin-coated plates (FBN), stained with FITC-phalloidin for the f-actin component of the cytoskeleton (green), p-Cofilin (red), and DAPI-stained nuclei (blue). Scale bar 20 μm. (**B**) Western blot analysis showing the expression levels of Cofilin, phospho-Cofilin, actin, and GAPDH following fibronectin treatment. The histogram shows the expression ratio of phospho-cofilin under the treated conditions relative to the CTR sample. ** *p*-value < 0.01, *n* = 3. (**C**) Predicted functional associations between proteins of interest, with a focus on “ECM structural constituents” (GO term) and cofilin

Brightfield microscopy and immunofluorescence (Fig. 8A) showed that fibronectin-treated RT4-D6P2T cells acquired an elongated, spindle-shaped phenotype compared to the polygonal morphology observed in controls. These morphological changes are consistent with fibronectin-associated ECM signaling and may reflect Schwann cell plasticity in vitro, linking the observed phenotype to a pro-regenerative state.

To investigate whether this morphological shift was accompanied by modulation of the cofilin pathway, total protein lysates were collected and analyzed by western blot for total cofilin, phospho-cofilin, actin, and GAPDH (Fig. 8B). Total cofilin and actin levels remained unchanged relative to controls; in contrast, phospho-cofilin levels were significantly reduced in fibronectin-treated cells, indicating a shift in the cofilin phosphorylation balance toward the active, dephosphorylated form. Since cofilin activity is regulated by the phosphorylation status of Ser3, with dephosphorylation enabling actin filament severing and promoting cytoskeletal dynamics, these findings are consistent with fibronectin-driven ECM signaling and suggest cofilin activation and actin remodeling in Schwann cells.

Overall, these data establish a mechanistic link between fibronectin-enriched ECM remodeling and the cofilin-actin axis, supporting the relevance of the proteomic signatures identified in human nerve tissue and the STRING-predicted protein-protein interaction network for the identified ECM and cytoskeletal proteins (Fig. 8C).

## Discussion

Peripheral neuropathies are often characterized by progressive axonal loss and subsequent disability in patients, representing a significant clinical challenge. Although the PNS possesses an intrinsic regenerative capacity, recovery typically remains incomplete, resulting in chronic sensory and motor impairments. Most studies have traditionally focused on neurons and Schwann cells, but growing evidence highlights the critical role of ECM in creating a supportive microenvironment for nerve regeneration. The ECM is essential for maintaining tissue homeostasis, regulating cellular behavior, and ensuring structural support. Altered ECM synthesis, degradation, or remodeling is a common feature of various diseases [21], including peripheral neuropathies [14, 22–24].

Sural nerve biopsies provide valuable information on the histopathological and molecular conditions associated with different axonal neuropathies, including acute axonal, regenerating axonal, and non-regenerating axonal neuropathies [25]. These conditions reflect distinct stages of nerve damage and disease progression, involving specific ECM remodeling processes that drive progression from acute damage to either regeneration or severe, non-regenerative chronic neuropathy. In this study, we employed a proteomic approach to characterize the matrisome composition in human nerve tissue across these disease stages. Despite inherently limitations arising from the use of archival FFPE sural nerve biopsies with very small tissue samples and data-dependent acquisition on a first-generation Q Exactive Orbitrap instrument available at the time of the study, the analysis successfully provided a robust and reproducible characterization of disease-related ECM remodeling. These results offer an important overview of the human sural nerve matrisome across various neuropathological conditions and lay a foundation that can be expanded in future research with next-generation mass spectrometers and DIA-based proteomics, which are likely to improve both proteome coverage and sensitivity.

We aimed to define the ECM dynamics that facilitate or hinder nerve regeneration in cases of acute axonal injury, regenerating axonal neuropathy, and non-regenerating axonal neuropathy. Notably, the proteins that were overrepresented in the control group (Group 1) were predominantly associated with Cytoskeleton organization. Specifically, Cellular components of Postsynaptic intermediate filament cytoskeleton, Myelin sheath, Axon, and Cytoskeleton describe a typical/physiological cellular architecture. Compared to control nerves, the ECM was the most significantly modulated molecular network across the different groups. GO analysis describes a microenvironment scenario characterized by decreased cellularity and a relative expansion of the collagen-rich extracellular matrix.

Acute axonal damage significantly alters the ECM composition. In the early stages of nerve damage, axonal degeneration initiates a cascade of cellular and humoral responses, leading to disruption of the blood–nerve barrier [26] and infiltration of blood-derived molecules, such as fibrinogen. This process contributes to the formation of a provisional ECM that supports Schwann cell proliferation and differentiation. This pathological scenario, characterized by elevated fibrinogen and compromised blood–nerve barrier integrity, is observed in patients of Group 2 with acute neuropathy and active axonal degeneration. A mature ECM rich in fibronectin is essential for efficient human nerve regeneration [14, 27–28]. Studies using artificial nerve conduits have demonstrated that fibronectin, combined with alginate matrices, enhances Schwann cell viability and growth in vitro and axonal regeneration in vivo [29]. These findings highlight the crucial role of fibronectin in creating a permissive environment by promoting cell adhesion, Schwann cell migration, and axonal outgrowth [14]. Interestingly, among the key molecular players in this process, we found Cofilin, an actin-binding protein that regulates actin filament turnover and cytoskeleton dynamics [30–32]. Cofilin plays a critical role in actin cytoskeletal remodeling, which is essential for axonal growth and regeneration in the PNS and CNS [33–38]. Immunofluorescence analysis revealed distinct Cofilin expression patterns in axonal neuropathies. In acute axonal neuropathies, Cofilin was elevated, suggesting an active role in axonal degeneration and subsequent regeneration. This expression supports the increased actin dynamics required for axonal regeneration. In regenerating nerves, Cofilin expression was moderate, likely reflecting stabilized cytoskeletal remodeling. Conversely, in human non-regenerating nerves, Cofilin expression is reduced, suggesting a decreased actin turnover and impaired regenerative capacity. Cofilin’s activity is further modulated by its phosphorylation state [39–40], and impaired activity can reduce neurite outgrowth and axonal regeneration. Our analysis using antibodies against p-Cofilin shows decreased expression in non-regenerating human nerves, further highlighting the regulatory role of p-Cofilin in axonal injury and repair. Investigation of interactions with upregulated ECM proteins in Group 2, using the STRING platform, revealed a functional link between Cofilin and this network, as well as its direct interaction with fibronectin (Fig. 4). Cofilin’s actin activity enables cytoskeletal remodeling and Schwann cell myelination [41–42], while fibronectin supports axonal growth by promoting Schwann cell migration and axonal adhesion, suggesting their complementary role in axonal regeneration. While direct experimental evidence linking fibronectin to Cofilin activity in peripheral nerves is limited, studies in other cellular systems indicate a functional connection via the integrin–FAK–Rho signaling cascade [43–44]. Our findings show that fibronectin-mediated ECM signaling modulates cofilin activity in Schwann cells by reducing its phosphorylation, thereby shifting the balance toward the active, dephosphorylated form. This increase in cofilin activity is expected to enhance actin filament turnover and cytoskeletal remodeling, both of which are essential for Schwann cell differentiation and myelination. In affected nerves, a primordial/transient ECM establishes a microenvironment favorable to nerve regeneration. If regenerative conditions are met, this early ECM remodels into a mature fibronectin-rich matrix, which is crucial for Schwann cell differentiation and axonal regrowth [13]. In this respect, our proteomic analysis showed a prominent matrix remodeling in Group 3 compared to Group 1, which was instead enriched with neuronal cytoskeletal proteins. Additionally, Group 3 exhibited reduced levels of proteins whose decreased abundance is consistent with the formation of a specialized, permissive microenvironment, including HSPA1A (Hsp72), Serum amyloid P-component protein (APCS), Apolipoprotein A1 (APOA1), Serotransferrin (TF), Inter-alpha-trypsin inhibitor heavy chain 1 (ITIH1), Inter-alpha-trypsin inhibitor heavy chain 2 (ITIH2), and Complement C3.

Hsp72 is known to protect neurons during acute stress [45]. Its lower expression in regenerating nerves indicates a transition from an acute damage state to a survival/regenerative state that supports axonal elongation. Similarly, complement C3 is upregulated in acutely damaged nerves to facilitate the initial clearance of debris [46, 47]. However, as confirmed also by our proteomic analysis, it should be downregulated during regeneration to prevent excessive C3b accumulation, which inhibits neurite outgrowth. Moreover, our proteomic analysis revealed low levels of the serum proteins APOA1 and TF, which are essential for lipid and iron transport, as well as oxidative protection [48]. These findings suggest the re-establishment of a selective blood–nerve barrier that minimizes oxidative stress on axonal growth. Finally, the decreased abundance of ITIH1 and ITIH2, the members of the inter-alpha-trypsin inhibitor family involved in matrix stabilization and protease inhibition [49], along with APCS, a protein that stabilizes cellular debris in the ECM [50–51], suggests reduced ECM stabilization.

Overall, these changes indicate a shift toward a more dynamic and permissive scaffold for nerve regeneration.

In conclusion, our work on the ECM composition of human sural nerve biopsies has revealed significant differences across the three distinct neuropathological conditions: acute axonal damage, regenerating axonal neuropathy, and non-regenerating axonal neuropathy. We obtained insights into the mechanisms underlying nerve damage and regeneration, revealing that acute axonal degeneration is characterized by a specific ECM profile that may facilitate the initial response to nerve damage and is crucial for repair processes essential for recovery. In regenerating axonal neuropathy, the ECM plays a supportive role, fostering axonal regeneration and promoting functional recovery. Conversely, non-regenerating axonal neuropathy shows alterations in ECM composition, determining the failure of nerve repair mechanisms with consequent persistence of debilitating symptoms. Overall, our findings show that ECM remodeling is a key regulated aspect of peripheral nerve regeneration and provide a molecular basis that could guide future mechanistic research and the development of therapies targeting the injury microenvironment.

## Supplementary Information

Below is the link to the electronic supplementary material.

Supplementary Material 1.

## Data Availability

The mass spectrometry proteomics data have been deposited to the ProteomeXchange Consortium via the PRIDE partner repository [52] with the dataset identifier PXD068740. The analyzed matrices are provided in Supplementary Table 1.
